# High USP4 mRNA is associated with an HPV-positive status in head and neck squamous cell carcinoma patients

**DOI:** 10.1007/s00432-023-04872-2

**Published:** 2023-06-12

**Authors:** Alexandra Scheiflinger, Sega Al-Gboore, Bernhard J. Jank, Faris Brkic, Lorenz Kadletz-Wanke, Lukas Kenner, Gregor Heiduschka, Julia Schnoell

**Affiliations:** 1 Department of Otorhinolaryngology, Head and Neck Surgery, Medical University of Vienna, Waehringer Guertel 18-20, 1090 Vienna, Austria; 2 Department of Pathology, Medical University of Vienna, Vienna, Austria; 3 Christian Doppler Laboratory for Applied Metabolomics, Vienna, Austria; 4 Unit of Laboratory Animal Pathology, University of Veterinary Medicine, Vienna, Austria; 5 CBmed GmbH - Center for Biomarker Research in Medicine, Graz, Styria Austria

**Keywords:** USP4, Prognosis, Biomarker, Head and neck, Squamous cell carcinoma, Cancer

## Abstract

**Introduction:**

Head and neck squamous cell carcinoma (HNSCC) is among the most common cancers in the world with a low survival rate and common diagnosis at late stages. Deubiquitination of proteins is involved in tumor growth, metastasis, apoptosis, and immunosuppressive pathways. The impact of the ubiquitin-specific protease (USP4) on survival was only scarcely investigated so far. The goal of our research was to analyze the association of USP4 expression with prognosis and clinicopathological features in HNSCC.

**Methods:**

USP4 mRNA levels were derived from The Cancer Genome Atlas (TCGA) for a cohort of 510 patients. Protein expression of USP4 was analyzed by immunohistochemistry in a second cohort of 113 patients. Associations between USP4 levels and overall survival, disease-free survival and clinicopathological data were analyzed.

**Results:**

High levels of USP4 mRNA were associated with prolonged overall survival in univariable analysis. There was no more association with survival after correction for the confounders HPV, stage and smoker status. High USP4 mRNA levels were linked to a lower T-stage, the patient’s age at diagnosis, and a positive HPV status. USP4 protein levels were not associated with prognosis or other features.

**Conclusion:**

Since high USP4 mRNA was not an independent prognostic marker, we assume that the association is a result of the correlation of high USP4 mRNA with an HPV-positive status. Therefore, further investigation of USP4 mRNA and its association with the HPV status of HNSCC patients is warranted.

## Introduction

Head and neck squamous cell carcinoma (HNSCC) is one of the leading causes of cancer-associated death worldwide, with a high morbidity and only slight improvement of quality of life and life expectancy (Johnson et al. 2020). To reduce mortality and improve quality of life for HNSCC patients, treatment options need to be improved. In the recurrent and metastatic setting, the use of nivolumab and pembrolizumab have greatly improved therapeutic options (Ferris et al. 2016; Cohen et al. 2019; Burtness et al. 2019). However, overall survival remains low and the increase in survival rates is partly due to the emergence of HPV-positive HNSCC (Johnson et al. 2020; Chaturvedi et al. 2011). To date, only few prognostic factors have been identified (Panarese et al. 2019). Therefore, the aim of this study was to evaluate mRNA and protein expression of USP4 in HNSCC.

The ubiquitin–proteasome system is largely responsible for the balance between intracellular protein degradation and protein synthesis (He et al. 2017; Lim et al. 2013; Poondla et al. 2019). Aberrant expression of deubiquitinating enzymes (DUBs) has been implicated in the development of many pathologies. Particularly in cancer, malfunction of the ubiquitin–proteasome system has been linked to tumor formation and tumor metastasis. Crucial cancer-associated processes such as tumor growth, apoptosis, metastasis and chemoresistance are influenced by DUBs (Mennerich et al. 2019; Tanguturi et al. 2020; Poondla et al. 2019).

Ubiquitin-specific protease 4 (USP4) is a member of the DUB subfamily of cysteine proteases (Hu et al. 2021) and plays an essential physiological role by influencing DNA repair, cell growth, differentiation and immune response (S. Li et al. 2021). USP4 deubiquitinates a wide range of well-known target proteins thereby influencing important pathways such as TGF-beta, p53 or WNT/β-Catenin signaling (Hu et al. 2021; S. Li et al. 2021). Specifically, USP4 is indispensable for cellular homeostasis and balancing of the intracellular protein synthesis. Hence USP4 levels are strictly controlled and monitored under physiologic conditions (Hu et al. 2021). Frederick et al. were the first to note a reduced expression of USP4 in lung cancer relative to normal tissue samples (Frederick et al. 1998). Since then, alterations in USP4 expression were found in several cancer entities (Li et al. 2016; Xing et al. 2016; Wang et al. 2020; Zhong et al. 2018; Yao et al. 2017) including an upregulation in HNSCC (Hou et al. 2013). Additionally, USP4 is associated with outcome and progression of several cancers (Tao and You 2022; Zhou et al. 2019; Hu et al. 2021; Yao et al. 2017; Zhong et al. 2018; Wang et al. 2020).

Because of their potential to evolve tumor diagnosis and therapy, ubiquitin-specific proteases were studied extensively in the past (Vlasschaert et al. 2015). In particular, our previous work on PSMD14 (Schnoell et al. 2022), another member of the DUB system prompted us to investigate the role of USP4 in HNSCC. In this study, we aimed to elucidate USP4 mRNA and protein expression levels in relation to survival outcome and the association with clinicopathological aspects of HNSCC patients.

## Patients and methods

### The cancer genome atlas dataset (TCGA)

Patient data were drawn from cBioportal.org “The Cancer Genome Atlas (TCGA), Firehose Legacy” and supplemented with “TCGA, PanCancer Atlas” (Liu et al. 2018) and “TCGA, Nature 2015” (Lawrence et al. 2015) as described before by Schnoell et al. (2022). We included a total of 510 patients, who were treated between 1978 and 2012. Since perioperative complications could not be excluded, patients with an overall survival of less than two months were excluded from the panel, as were patients with incomplete survival records or missing mRNA data. The HPV status was determined by RNA sequencing of E6 and E7. Patients with over 1000 mapped RNA Seq reads were considered HPV positive (Liu et al. 2018). USP4 mRNA levels (RNA Seq V2 RSEM) were collected from cBioportal.org and classified as low or high expression based on a *z*-score > 0 criterion for high expression.

### The tissue microarray (TMA) dataset

For the second patient cohort, a tissue microarray (TMA) database encompassing 113 HNSCC patients was probed for USP4 protein levels using immunohistochemistry. Included patients were diagnosed with HNSCC between 2002 and 2012 and all treated with surgery and post-operative radio(chemo)therapy. The HPV status was assessed by in situ hybridization. The TMA was generated by using a Galileo TMA CK Series-HTS Tissue computer assisted TMA Microarray platform (Integrated Systems Engineering Srl, Milan, Italy). Briefly, three cylindrical cores (2 mm in diameter) from formalin-fixed, paraffin-embedded HNSCC tissue were included from each patient. Conformation of histology was done by hematoxylin–eosin staining. The study was authorized by the Medical University of Vienna's ethical committee (EK1262/2019).

### Immunohistochemistry

Immunohistochemistry was done following the manufacturer's protocol of the Ultra Vision Quanto Detection System HRP (Thermo Fisher Scientific, Waltham, MA, USA) with the following modifications (Schnoell et al. 2022). Briefly, samples were deparaffinized, rehydrated and incubated in H_2_O_2_ to suppress endogenous peroxidase activity. Samples were heated in EDTA in a microwave for antigen retrieval. Slides were then incubated with the Ultra V Block and afterwards with the USP4 primary antibody (1:500, HPA018499-25UL, Sigma Aldrich, Darmstadt, Germany) for 60 min at room temperature. Next, the primary antibody enhancer and horseradish peroxidase enhancer were applied. Stains were developed using DAB Quanto Detection System (Thermo Fisher Scientific, Waltham, MA, USA). Samples were washed with deionized water and counterstained with Hematoxylin Gill II (Merck, Darmstadt, Germany). Imaging was done with a PANNORAMIC Digital Slide Scanner and the open-source software QuPath (Edinburgh, Scotland). The *h*-score was calculated by combining the percentage of stained cells and the staining intensity (3 × % of cells with high positive staining + 2 × % of cells with moderately positive staining + 1 × % of cells with low positive staining). Staining was defined as high at a *h*-score cutoff value > 70 using the median *h*-score as reference.

### Statistical analysis

STATA software (Stata Corp, Houston TX, USA, SCR_012763) and GraphPad Prism Software (GraphPad Prism Software, Inc., La Jolla, CA, USA, SCR_002798) were used to analyze all collected information. Continuous variables were evaluated with median values and 25th and 75th percentiles, while categorical data were reported in absolute and relative frequencies. The median observation was calculated as described by Schemper and Smith (1996). Overall survival and disease-free survival were calculated from time of initial pathological diagnosis until death or tumor recurrence, respectively. To visualize overall survival and disease-free survival, Kaplan–Meier curves were created. To evaluate the relation of clinicopathological features with USP4 expression, correlation analysis was performed using Fisher’s exact test and Chi^2^ test. Investigation of the association of USP4 mRNA and protein expression with overall survival and disease-free survival was done using log-rank test and uni- and multivariable analysis using the Cox proportional hazard regression model. A *p* value ≤ 0.05 was considered statistically significant.

## Results

### TCGA dataset: baseline characteristics

The TCGA dataset comprised 510 patients, 132 (26%) females and 378 (74%) males. Their characteristics are shown in Table 1. Median age at diagnose was 60.5 years (53–68). The majority of patients suffered from carcinoma of the oral cavity (60%), were diagnosed at stage IV (55%) and were HPV-negative (81%). The median observation time was 34.8 (21.6–55.2) months. The median overall survival was 56.9 (16.3–153.7) months, and the median disease-free survival was 56.4 (12.8–211.0) months.

**Table 1:** Baseline characteristics of the primary (TCGA) and secondary (TMA) HNSCC dataset

	Primary dataset (TCGA)		Secondary dataset (TMA)	
	Number of patients	Percentage (%)	Number of patients	Percentage (%)
*Gender*				
Female	132	26	27	24
Male	378	74	86	76
*Age*				
< 60	231	45	61	54
≥ 60	279	55	52	46
*Primary*				
Oral Cavity	307	60	30	27
Oropharynx	79	16	51	45
Hypopharynx	10	2	21	19
Larynx	114	22	11	10
*HPV*				
Negative	413	81	87	78
Positive	76	15	24	22
x	21	4	0	0
*T Stage*				
1	34	7	21	19
2	148	29	60	53
3	133	26	20	18
4	180	35	12	11
x	15	3	0	0
*N Stage*				
0	237	5	23	20
1	81	16	29	26
2	162	32	61	54
3	9	2	0	0
x	21	4	0	0
*M Stage*				
0	480	94	79	70
1	6	1	0	0
x	24	5	34	30
*Staging*				
I	20	4	2	2
II	94	18	15	13
III	103	20	29	26
IV	280	55	67	59
x	13	3	0	0
*Smoker*				
Never/Ex	323	63	96	85
Active	173	34	15	13
x	14	3	2	2
*Radiotherapy*				
No	86	17	0	0
Adjuvant	189	37	113	100
Primary	18	4	0	0
Neoadjuvant	6	1	0	0
x	211	41	0	0
*Pharmaceutical therapy*				
No	129	25	95	84
Adjuvant	113	22	18	16
Primary	4	1	0	0
Neoadjuvant	8	2	0	0
x	256	50	0	0

### TCGA dataset: association of USP4 mRNA levels with prognosis

**Fig. 1:**
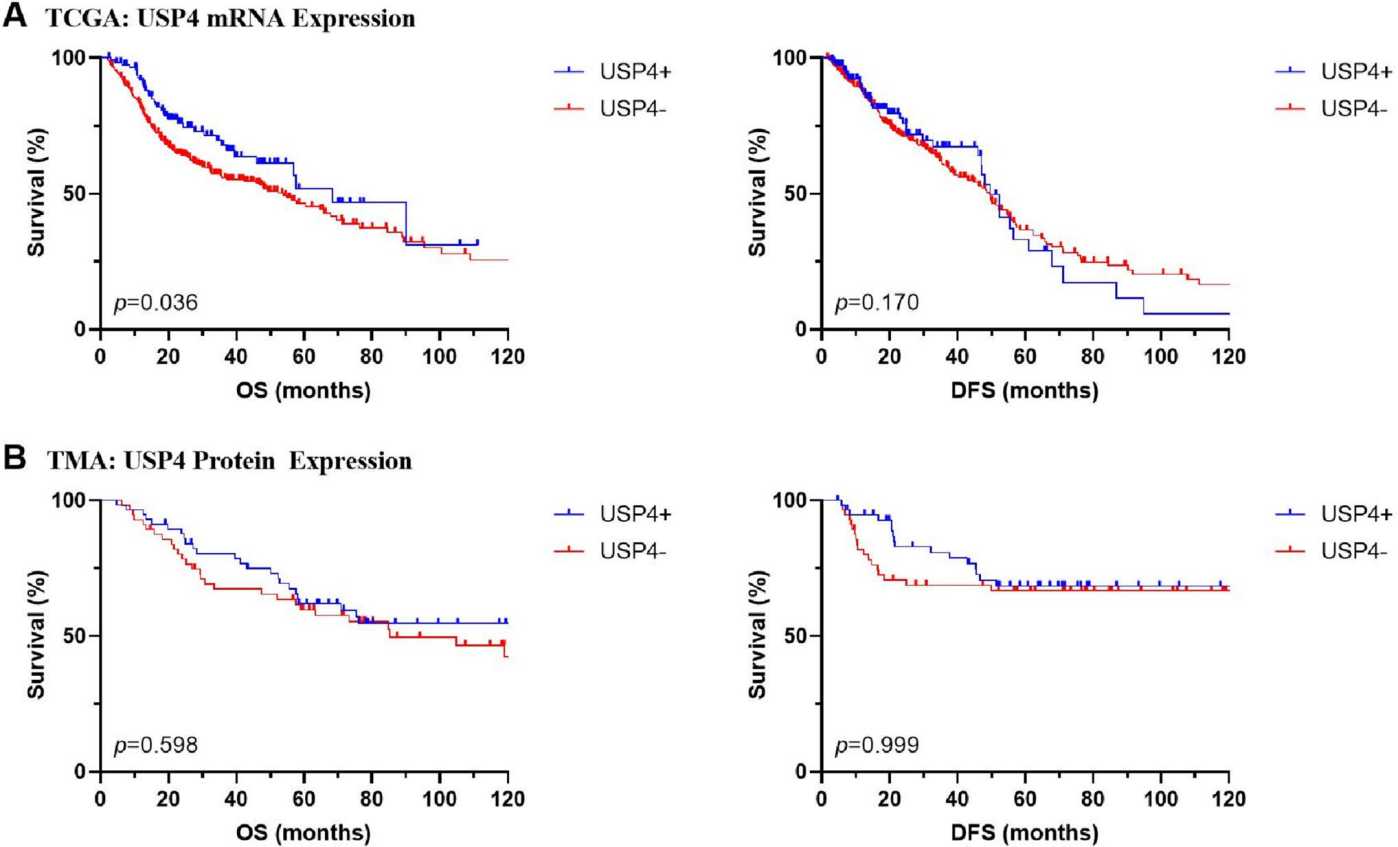
Kaplan–Meier survival curves of overall survival (OS) and disease-free survival (DFS). **A** TCGA: OS and DFS stratified by USP4 mRNA expression, **B** TMA: OS and DFS stratified by USP4 protein expression. *p:* log-rank *p* value; USP4 + : high; USP4−: low

Analysis of survival data is presented in Table 2 and Fig. 1. One-hundred and fifteen (23%) patients showed high USP4 mRNA expression levels. Patients with high USP4 mRNA expression showed improved overall survival outcomes compared to the low expression group (53.9 vs. 68.4 months; *p* = 0.036). In univariable analysis, patients with high USP4 expression were at lower risk of death (HR 0.68, 95% CI 0.48–0.98, *p* = 0.037). Subsequent evaluation of the continuous mRNA *z*-scores identified a reduced mortality risk for the group with high mRNA expression (HR 0.81, 95% CI 0.69–0.96, *p* = 0.013) in univariable analysis. Multivariable analysis, corrected for disease stage, smoker and HPV status, did not show an association of overall survival or disease-free survival with USP4 mRNA levels. Disease-free survival was not correlated to high USP4 mRNA expression (56.4 vs. 56.5 months; *p* = 0.170) nor the risk of recurrence (HR 0.78, 95% CI 0.55–1.11, *p* = 0.172; continuous: HR 0.88, 95% CI 0.75–1.04, *p* = 0.149).

**Table 2:** Uni- and multivariable analysis of overall survival and disease-free survival of USP4 mRNA (TCGA) and protein (TMA) levels

	Univariable			Multivariable		
	HR	95% CI	*p* value	HR	95% CI	*p* value
*Overall Survival*						
TCGA						
USP4 mRNA *high vs. low*	0.68	0.48–0.98	**0.037**	0.86	0.59–1.27	0.460
USP4 mRNA *z*-score	0.81	0.69–0.96	**0.013**	0.88	0.73–1.06	0.166
TMA						
USP4 protein *high vs. low*	0.87	0.51–1.47	0.599	0.81	0.47–1.39	0.448
USP4 protein *h*-score	1.00	0.99–1.01	0.975	0.99	0.99–1.01	0.945
*Disease-Free Survival*						
TCGA						
USP4 mRNA high vs. low	0.78	0.55–1.11	0.172	0.89	0.61–1.32	0.574
USP4 mRNA *z-*score	0.88	0.75–1.04	0.149	0.94	0.77–1.13	0.496
TMA						
USP4 protein high vs. low	1.00	0.53–1.89	1.000	0.91	0.48–1.73	0.776
USP4 protein *h*-score	1.00	0.99–1.02	0.498	1.00	0.99–1.02	0.744

Multivariable analysis was corrected for staging, HPV and smoker status. A *p* value below 0.050 was considered significant (bold). HR hazard ratio. CI confidence interval

**Fig. 2:**
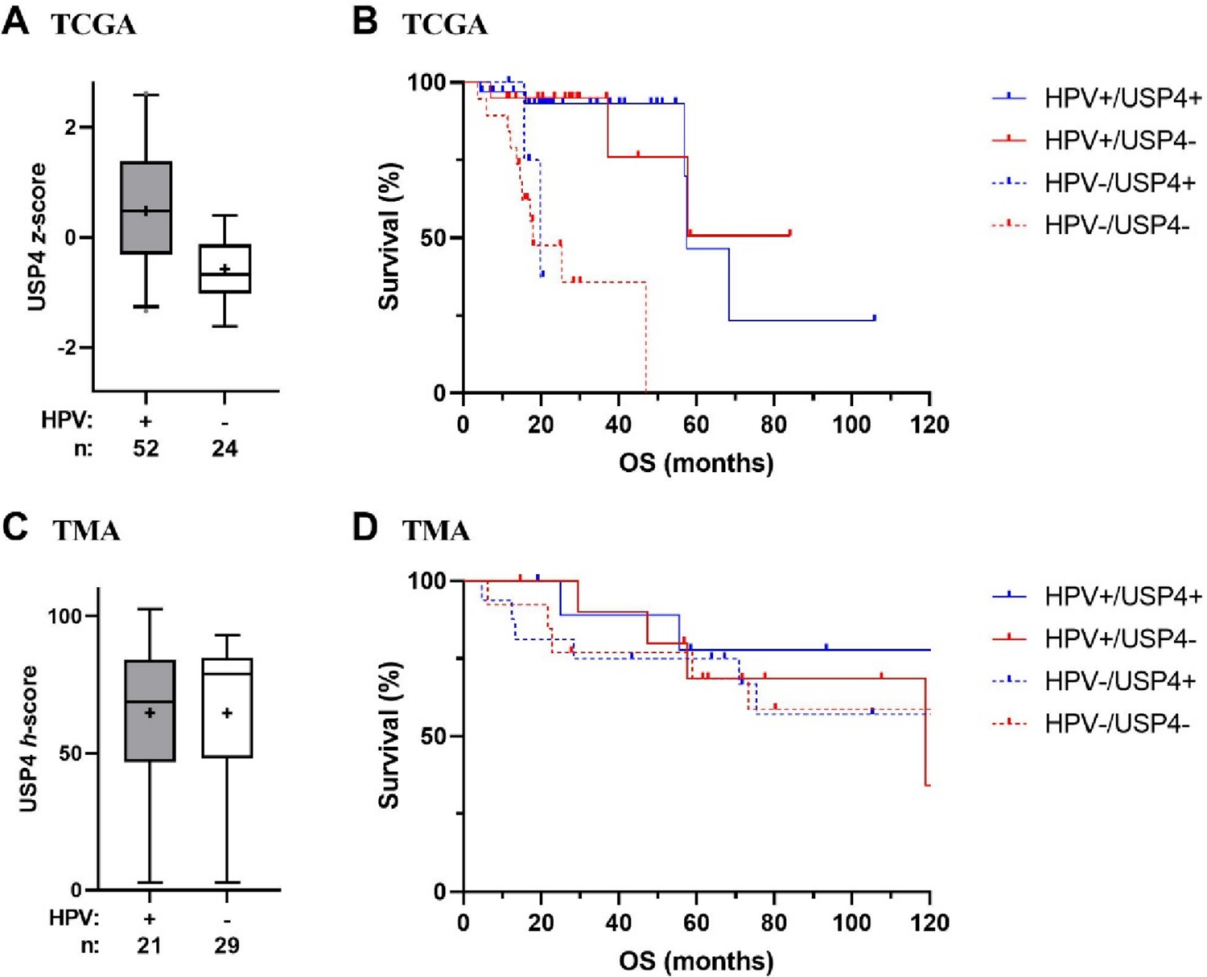
Subgroup analysis of patients with oropharyngeal squamous cell carcinoma. **A** TCGA: USP4 mRNA expression (*z*-score) stratified by the HPV status. **B** TCGA: overall survival stratified by USP4 mRNA expression and HPV status. **C** TMA: USP4 protein expression (*h*-score) stratified by the HPV status. **D** TMA: overall survival stratified by USP4 mRNA expression and HPV status. HPV + : positive; HPV-: negative; USP4 + : high; USP4−: low

The role of USP4 mRNA as a prognostic marker was further analyzed in the subgroups of HPV-positive and -negative patients. No association with overall or disease-free survival was found. Kaplan–Meier curves for the overall survival of patients with oropharyngeal carcinoma stratified by the HPV status and USP4 mRNA expression are shown in Fig. 2B.

### TCGA dataset: association of USP4 mRNA levels with clinicopathological data

To evaluate the association of USP4 mRNA expression with clinicopathological data, correlation analysis using Fisher’s exact or Chi^2^ test was performed (Table 3). High expression of USP4 mRNA was associated with lower age (< 60 years, *p* < 0.001), a lower T-stage (T1-2, *p* = 0.049) and an HPV-positive status (*p* < 0.001). The median USP4 *z-*score was 0.25 (− 0.49 to 1.10) in HPV-positive patients and − 0.71 (− 1.19to − 0.22) in HPV-negative patients. In oropharyngeal carcinoma patients the median *z-*score was 0.48 (− 0.31 to 1.37) in HPV-positive patients and − 0.68 (− 1.01to − 0.14) in HPV-negative patients (Fig. 2A). In oral carcinoma patients the median *z*-score was 0.10 (− 0.49 to 0.58) for HPV-positive patients and -0.76 (− 1.22 to − 0.27) for HPV-negative patients. The mRNA levels were not investigated separately for other locations of the primary due to the low number of HPV-positive patients.

**Table 3:** Correlation analysis of USP4 mRNA (TCGA) and protein level (TMA)

	Primary dataset (TCGA)			Secondary dataset (TMA)		
	USP4 mRNA			USP4 protein		
	Low	High	*p* value	Low	High	*p* value
*Sex*						
Female	97 (25%)	35 (30%)		15 (27%)	12 (21%)	
Male	298 (75%)	80 (70%)	0.205	41 (73%)	45 (79%)	0.475
*Age*						
< 60	161 (41%)	70 (61%)		31 (55%)	30 (53%)	
≥ 60	234 (59%)	45 (39%)	** < 0.001**	25 (45%)	27 (47%)	0.771
*T Stage*						
T1-2	132 (35%)	50 (45%)		15 (27%)	17 (30%)	
T3-4	251 (66%)	62 (55%)	**0.049**	41 (73%)	40 (70%)	0.720
*N Stage*						
N0	184 (49%)	53 (47%)		14 (25%)	10 (18%)	
N1-3	192 (51%)	60 (53%)	0.704	42 (75%)	47 (83%)	0.333
*Staging*						
I–II	88 (23%)	26 (23%)		9 (16%)	8 (14%)	
III–IV	296 (77%)	87 (77%)	0.984	47 (84%)	49 (86%)	0.762
*HPV*						
Negative	345 (91%)	68 (62%)		42 (76%)	45 (80%)	
Positive	34 (9%)	42 (38%)	** < 0.001**	13 (24%)	11 (20%)	0.609
*Smoker*						
Never/ex	245 (64%)	78 (69%)		27 (48%)	23 (40%)	
Active	138 (36%)	35 (31%)	0.982	29 (52%)	34 (60%)	0.400

Significance was reached at a *p* value of 0.050 and below (bold)

### TMA dataset: association of USP4 protein levels with outcome and clinicopathological data

A total of 113 patients, 27 females (24%) and 86 males (76%), at a median age of 59 (53–63) years were included in the TMA cohort. Most patients were HPV-negative (78%), nonsmokers (53%) and predominantly had a carcinoma of the oropharyngeal region (45%). Median observation time was 114.8 (76.3–141.7) months with a median overall survival time of 119.0 (30.72–not reached) months and a disease-free survival period of 156.8 (32.2–not reached) months. All patients were treated with surgery and post-operative radiotherapy.

**Fig. 3:**
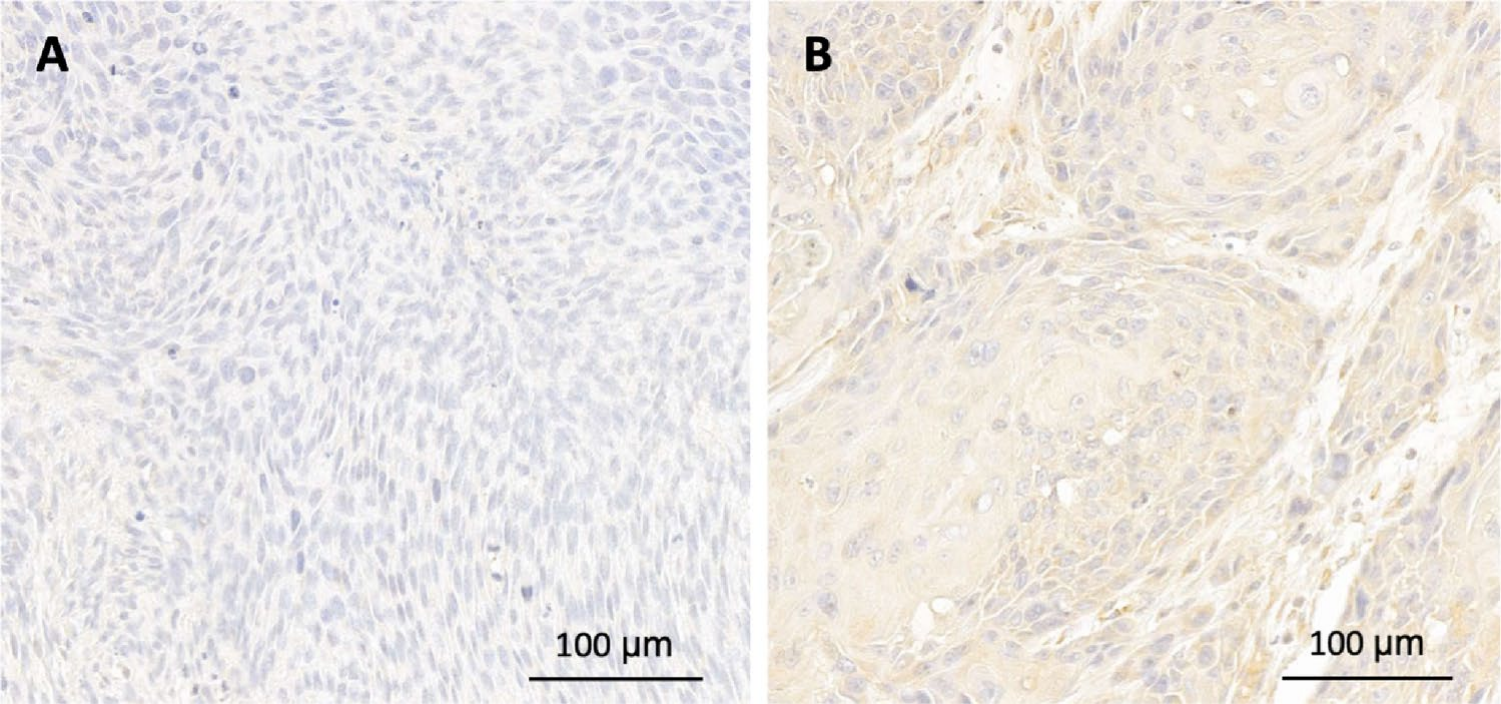
Tissue samples showing low (**A**) or high (**B**) USP4 protein expression in HNSCC samples analyzed by immunohistochemistry. *h*-score cutoff > 70 for high expression

Protein expression was evaluated using immunohistochemistry and scored using the *h*-score. The median *h*-score of USP4 was 70.2 (47.4–82.8). The cutoff for high expression was > 70, using the median expression level as guidance. Thus, 57 patients (50%) showed high expression of USP4 demonstrated in Fig. 3. Kaplan–Meier curves were calculated (Fig. 1). Patients with high USP4 protein levels showed a trend for a prolonged overall survival (13.6 vs. 7.1, *p* = 0.598) and disease-free survival (not reached-12.7 months, *p* = 0.999). However, these results did not reach statistical significance. Further regression analysis did not show any association with the risk of death or recurrence in uni- or multivariable analysis (Table 2). There was no observed correlation with clinicopathological data (Table 3).

To further investigate the association of USP4 with the HPV status, subgroup analysis was performed. The median *h*-score for all HPV-positive patients was 68.5 (47.5–84.1) and 72.5 (47.3–83.0) for HPV-negative patients. In oropharyngeal cancer patients the median *h*-score was 68.6 (47.4–79.4) for HPV-positive patients and 78.8 (48.9–84.5) for HPV-negative patients (Fig. 2C). Due to the low number of HPV-positive patients, other locations of the primary tumor were not analyzed. Subgroup analysis of overall and disease-free survival of HPV-positive and -negative patients showed no association with outcome. Kaplan–Meier curves for the overall survival stratified by the HPV status und USP4 protein expression of oropharyngeal carcinoma patients are shown in Fig. 2D.

## Discussion

HNSCC remains one of the most common carcinomas worldwide. The majority of patients are diagnosed at late disease stages with poor survival rates. As the needed multimodal therapy has many consequences and affects quality of life, better stratification of high and low risk patients would be beneficial. Thus, biomarkers are required to improve therapeutic decisions (Johnson et al. 2020). The deubiquitinating enzyme USP4 is associated with outcome in several carcinomas (Tao and You 2022; Zhou et al. 2019; Zhong et al. 2018; Wang et al. 2020; Yao et al. 2017). In HNSCC, USP4 is upregulated compared to non-cancerous tissue and has tumor suppressor qualities in vitro (Hou et al. 2013). However, the role of USP4 as a prognostic marker in HNSCC has not been investigated so far.

Our study aimed to investigate the association of USP4 protein and mRNA levels with HNSCC prognosis and clinicopathological data. First, mRNA expression data were derived from the TCGA dataset. Twenty-three percent of patients showed high mRNA expression. In survival analysis, high USP4 mRNA proved to be a marker for prolonged overall survival (53.9 vs. 68.4 months; log-rank *p* = 0.036) and a lower risk of death in univariable analysis (grouped: HR 0.68, 95% CI 0.48–0.98, *p* = 0.037, continuous: HR 0.81, 95% CI 0.69–0.96, *p* = 0.013). However, there was no more association with survival in multivariable analysis after correction for confounders (staging, HPV and smoker status). To further elucidate the change after multivariable analysis, we next investigated the correlation with clinicopathological data. This revealed associations of USP4 mRNA levels with T staging (*p* = 0.049) and HPV status (*p* < 0.001), but not with overall staging (*p* = 0.984) or smoker status (*p* = 0.982). HPV-negative patients show a worse outcome in oropharyngeal carcinoma (Paver et al. 2020), while the relevance for other sites is controversial (Tian et al. 2019; Zhu et al. 2019). Our survival analysis of the TCGA cohort showed that HPV was a positive predictive factor for a prolonged overall survival in the whole cohort (5.7 vs. 4.1 months, log-rank *p* = 0.002, univariable: HR 0.46, 95% CI 0.28–0.76 *p* = 0.002, multivariable adjusted for staging and smoker status: HR 0.43, 95% CI 0.26–0.72, *p* = 0.001). Therefore, we assume that the association of high USP4 mRNA levels with a better survival in the univariable analysis may be a result of the correlation of high USP4 mRNA levels with an HPV-positive status. In accordance with our results, Lohavanichbutr et al*.* showed that USP4 is downregulated in HPV-negative oropharyngeal cancer (Lohavanichbutr et al. 2009). Furthermore, the USP4 gene is an integration site for HPV16 in cervical and oropharyngeal squamous cell carcinoma, which may lead to alteration of gene expression (Díaz-Moreno et al. 2020). Hou et al. showed that USP4 has a tumor suppressor role in HNSCC in vitro (Hou et al. 2013). In other cancer entities, high USP4 mRNA was a positive prognostic marker in lung adenocarcinoma (Zhong et al. 2018) and a negative prognostic marker in glioblastoma patients (Zhou et al. 2019). Lung adenocarcinoma patients with a high USP4 status more commonly had no lymph node metastases (Zhong et al. 2018). However, in contrast to our results, USP4 prevailed as an independent prognostic factor for adenocarcinoma patients after correction for confounders in multivariable analysis of overall and disease-free survival (Zhong et al. 2018).

At protein level, the role of USP4 as a prognostic marker is controversial as well. High USP4 is associated with a worse outcome in gastric cancer (Tao and You 2022) and pancreatic cancer (Wang et al. 2020) and a better outcome in esophageal cancer (Yao et al. 2017). To elucidate whether the results of USP4 mRNA expression corresponded to protein levels in HNSCC patients, the association USP4 protein expression was analyzed in a secondary cohort. Tumor samples from a total of 113 patients (TMA dataset) were analyzed for USP4 protein levels semi-quantitively using immunohistochemistry. Patients with high USP4 protein levels showed a trend for an extended overall survival (13.6 vs. 7.1, *p* = 0.598) and disease-free survival (not reached vs. 12.7 months, *p* = 0.999). However, these results did not reach statistical significance. In cox regression analysis, USP4 protein levels (grouped and continuous) had no predictive power with respect to overall survival and or disease-free survival. Additional analysis of the association with clinicopathological data revealed no significant correlations. While it is reasonable to assume that mRNA and protein levels generally correlate with each other, post-translational mechanisms have the potential to alter protein expression (Liu et al. 2016). Furthermore, a selection bias may apply, since the second cohort (TMA) only contains patients who received surgery and post-operative radio(chemo)therapy, whereas the TCGA cohort also contains other treatment regimens.

Following additional limitations may have influenced the findings and conclusions of this study. A selection bias, leading to a skewed outcome might have been introduced through the retrospective study design. To minimize this risk, we included two separate cohorts. Although TMA cores were analyzed from several representative tumor areas, protein expression within other regions of the cancerous tissue might vary. To exclude type I and II errors due to a biased cutoff level of the categorical USP4 levels, we additionally analyzed the continuous *z*- and *h*-scores.

Altogether, this study showed that high USP4 mRNA is not an independent prognostic factor for a longer overall survival in HNSCC patients. The association with survival in univariable analysis may be due an association of high USP4 mRNA with HPV-positive patients. Therefore, further investigation of the role of USP4 in HPV-positive HNSCC is warranted. These findings were not validated in the second cohort at protein level.

## Data Availability

The datasets of this study are available from the corresponding author on reasonable request.
